# Involvement of Cannabinoid Signaling in Vincristine-Induced Gastrointestinal Dysmotility in the Rat

**DOI:** 10.3389/fphar.2017.00037

**Published:** 2017-02-06

**Authors:** Gema Vera, Ana E. López-Pérez, José A. Uranga, Rocío Girón, Ma Isabel Martín-Fontelles, Raquel Abalo

**Affiliations:** ^1^Área de Farmacología y Nutrición, Departamento de Ciencias Básicas de la Salud, Universidad Rey Juan CarlosAlcorcón, Spain; ^2^Unidad Asociada I+D+i del Instituto de Química Médica, Consejo Superior de Investigaciones CientíficasMadrid, Spain; ^3^Unidad Asociada I+D+i del Instituto de Investigación en Ciencias de la Alimentación, Consejo Superior de Investigaciones CientíficasMadrid, Spain; ^4^Grupo de Excelencia Investigadora URJC-Banco de Santander-Grupo Multidisciplinar de Investigación y Tratamiento del Dolor (i+DOL)Alcorcón, Spain; ^5^Unidad del Dolor, Servicio de Anestesia, Hospital General Universitario Gregorio MarañónMadrid, Spain; ^6^Área de Histología Humana y Anatomía Patológica, Departamento de Ciencias Básicas de la Salud, Universidad Rey Juan CarlosAlcorcón, Spain

**Keywords:** chemotherapy-induced adverse effects, cannabinoid, CB_1_ receptor, gastric emptying, radiology, rat, vincristine, ileus

## Abstract

**Background:** In different models of paralytic ileus, cannabinoid receptors are overexpressed and endogenous cannabinoids are massively released, contributing to gastrointestinal dysmotility. The antitumoral drug vincristine depresses gastrointestinal motility and a similar mechanism could participate in this effect. Therefore, our aim was to determine, using CB_1_ and CB_2_ antagonists, whether an increased endocannabinoid tone is involved in vincristine-induced gastrointestinal ileus.

**Methods:** First, we confirmed the effects of vincristine on the gut mucosa, by conventional histological techniques, and characterized its effects on motility, by radiographic means. Conscious male Wistar rats received an intraperitoneal injection of vincristine (0.1–0.5 mg/kg), and barium sulfate (2.5 ml; 2 g/ml) was intragastrically administered 0, 24, or 48 h later. Serial X-rays were obtained at different time-points (0–8 h) after contrast. X-rays were used to build motility curves for each gastrointestinal region and determine the size of stomach and caecum. Tissue samples were taken for histology 48 h after saline or vincristine (0.5 mg/kg). Second, AM251 (a CB_1_ receptor antagonist) and AM630 (a CB_2_ receptor antagonist) were used to determine if CB_1_ and/or CB_2_ receptors are involved in vincristine-induced gastrointestinal dysmotility.

**Key results:** Vincristine induced damage to the mucosa of ileum and colon and reduced gastrointestinal motor function at 0.5 mg/kg. The effect on motor function was particularly evident when the study started 24 h after administration. AM251, but not AM630, significantly prevented vincristine effect, particularly in the small intestine, when administered thrice. AM251 alone did not significantly alter gastrointestinal motility.

**Conclusions:** The fact that AM251, but not AM630, is capable of reducing the effect of vincristine suggests that, like in other experimental models of paralytic ileus, an increased cannabinoid tone develops and is at least partially responsible for the alterations induced by the antitumoral drug on gastrointestinal motor function. Thus, CB_1_ antagonists might be useful to prevent/treat ileus induced by vincristine.

## Introduction

Vincristine is a vinca alkaloid widely used in the treatment of hematological malignancies and solid tumors since the 1960's (Johnson et al., 1960; Bohannon et al., 1963). It is a cell cycle specific agent which blocks mitosis with metaphase arrest through disruption of the mitotic apparatus and it may affect several body systems (Rosenthal and Kaufman, 1974). The main side effect of vincristine is a dose dependent and cumulative peripheral neuropathy. Paresthesias, loss of tendon reflexes, and progressive weakness are the most common clinical features, although autonomic dysfunctions, including gastrointestinal disturbances, might occur (Rosenthal and Kaufman, 1974; Harris and Jackson, 1977; Chae et al., 1998; Wang et al., 2000). Indeed, gastrointestinal complications may be present in up to 30–40% of patients receiving vincristine and the earliest symptoms may include colicky abdominal pain, constipation, and adynamic or paralytic ileus as the major manifestations. Damage to the myenteric plexus by vinca alkaloids could be implicated in intestinal hypomotility (Smith, 1967; Kaneko et al., 2001; Peixoto Júnior et al., 2009). Since constipation is the most widely recognized manifestation, colonic motility has received the most attention. But patients treated with vincristine can also develop symptoms indicating dysmotility of the upper gastrointestinal tract, including anorexia, and nausea or even extreme symptoms such as paralytic ileus. In fact, paralytic ileus occurs in 3–12% of patients, and may be fatal in up to 30% of them (Toghill and Burke, 1970). However, the impact and mechanisms of vincristine on gastrointestinal motility have not been deeply studied in humans or animals.

The endocannabinoid system in the gastrointestinal tract has attracted much attention because both its activation and inhibition could be therapeutically useful depending on the circumstances (Abalo et al., 2012; Abalo and Martín-Fontelles, 2017; Salaga et al., 2017; Vera et al., 2017). Evidence is emerging that exogenous and endogenous cannabinoids have an important role in gastrointestinal physiopathology, such as gastrointestinal inflammation (Izzo and Camilleri, 2009). But cannabinoids mediate also other functions in the gut, such as gastroprotection and gastric secretion, gastrointestinal motility, ion transport, visceral sensation, and cell proliferation (Izzo and Sharkey, 2010). In this sense, plant-derived, endogenous, and synthetic cannabinoid receptor agonists reduced gastric emptying, upper gastrointestinal transit and colonic propulsion in rodents (Aviello et al., 2008; Izzo and Camilleri, 2009; Abalo et al., 2012; Vera et al., 2017), whereas cannabinoid receptor antagonists may increase gastrointestinal motility in experimental animals (Izzo et al., 1999) and cause diarrhea in humans (Waterlow and Chrisp, 2008).

There are functional, biochemical, and immunohistochemical evidences that alterations in the enteric endocannabinoid system contribute to causing paralytic ileus in animal models, and different strategies aimed at normalizing endocannabinoid levels were useful in these conditions. Actually, the inactivation of CB_1_ (Mascolo et al., 2002) or CB_1_ and CB_2_ receptors (Li et al., 2010) were useful in the treatment of paralytic ileus induced by acetic acid and lipopolysaccharide (LPS), respectively. Thus, cannabinoid antagonists may be powerful tools in the treatment of adynamic ileus of different origins.

So far, we have characterized the effect of different drugs in the gastrointestinal tract of experimental animals using radiographic methods, including antitumoral drugs, like cisplatin (Cabezos et al., 2008, 2010; Vera et al., 2014) and 5-fluorouracil (Abalo et al., 2016; McQuade et al., 2016), and cannabinoids (Abalo et al., 2009, 2010, 2011, 2015). We have even performed studies of the combined effects of antitumoral drugs and cannabinoids (Abalo et al., 2013, 2016). In these regards, we showed that the non-selective cannabinoid agonist WIN 55, 212-2 was not capable of improving cisplatin-induced gastrointestinal dysmotility, and even worsened it (Abalo et al., 2013), whereas at a non-psychoactive dose, it tended to reduce diarrhea associated to 5-fluorouracil treatment (Abalo et al., 2016). Thus, these techniques might be useful to study vincristine effects on gastrointestinal motor function, and the possible role of cannabinoid agents in them.

Therefore, the aims of this work were, using radiographic means: (1) To characterize the effect of the antitumoral drug vincristine on rat gastrointestinal motor function. (2) To determine whether the motor alterations induced by vincristine might be prevented by the CB_1_-selective cannabinoid antagonist AM251 and by the CB_2_-selective cannabinoid antagonist AM630. Some of the present results were communicated previously in abstract form (Vera et al., 2012).

## Materials and methods

The Ethical Committee at Universidad Rey Juan Carlos (URJC) and Hospital General Universitario Gregorio Marañón (HGUGM) approved the study. Experimental procedures were carried out in accordance with the recommendations of these Committee as well as with the EU directive for the protection of animals used for scientific purpose (2010/63/UE) and Spanish regulations (RD 109 53/2013).

### Animals

Male Wistar rats (350–400 g) were obtained from the Veterinary Unit of HGUGM (Madrid, Spain) or from Envigo (Barcelona, Spain) and housed (4/cage), at the Veterinary Units of HGUGM, or URJC, in standard transparent cages (60 × 40 × 20 cm), under environmentally controlled conditions (temperature = 20°C; humidity = 60%), with a 12 h light/12 h dark cycle. Animals had free access to standard laboratory rat chow (Harlan Laboratories Inc.) and tap water.

### Protocol

First, we characterized the effect of a single dose of vincristine on gastrointestinal architecture and motility by histological and radiographic means, respectively (see below). Rats received an acute intraperitoneal injection of vincristine (0.1 or 0.5 mg/kg) or saline (2–3 ml/kg). Alterations of gastrointestinal motility were measured immediately, 24 or 48 h after drug administration. Samples from ileum and colon were taken for conventional histology 48 h after saline or vincristine (0.5 mg/kg).

A second set of experiments was performed in order to determine whether the alterations induced by vincristine could be due to an increased cannabinoid tone and activation of CB_1_ or CB_2_ receptors. In these experiments, vincristine was administered 24 h prior to the radiographic analysis and adequate cannabinoid antagonists were tested as follows.

The cannabinoid CB_1_-selective antagonist AM251 (1 mg/kg), or its vehicle (1 ml/kg), was administered once (20 min before vincristine), twice (before and 24 h after vincristine), or thrice (before, 12 and 24 h after vincristine). Thereafter (24 h after vincristine injection), the radiographic analysis of gastrointestinal motor function was performed (see below).

In the remaining experiments, the CB_2_-selective antagonist AM630 (1 mg/kg), or its vehicle (1 ml/kg), was administered thrice (20 min before, and 12 and 24 h after vincristine) and gastrointestinal motor function was analyzed as described below. This group of experiments was performed at URJC, using animals from Envigo.

### Histology

Forty-eight hours after vincristine, samples were obtained from terminal ileum (at least 10 cm oral to the ileocaecal junction) and colon of 4–8 animals per experimental group, fixed in buffered 10% formalin and embedded in paraffin. Sections of 5 μm were stained with hematoxylin-eosin (HE) and studied under a Zeiss Axioskop 2 microscope equipped with the image analysis software package AxioVision 4.6. Samples were studied in duplicate under a 20x objective. Histological damage was evaluated using a numerical score of 0–3 assigned to each section considering general loss of mucosal architecture (graded 0–3, absent to severe) and extent of inflammatory cell infiltrate (graded 0–3, absent to transmural). The experimenter was blind to the treatment received by the rat from which the sample under analysis was obtained.

### Gastrointestinal motility evaluation

Gastrointestinal motor function was studied by radiographic methods as previously described (Cabezos et al., 2008). Thus, 2.5 ml of a suspension of barium sulfate (2 g/ml, temperature = 22°C) was administered *per os*. Experiments at HGUGM were performed with a Siemens (Siremobil Compact L, Erlangen, Germany) digital X-Ray apparatus (60 kV, 7 mA) and X-rays were captured with NPG Real DVD Studio II software. For the experiments at URJC, a CS2100 (Carestream Dental, Spain) digital X-ray apparatus (60 kV, 7 mA) was used, and X-rays were recorded on Carestream Dental T-MAT G/RA film (15 × 30 cm) housed in a cassette provided with regular intensifying screen; films were developed using a Kodak X-omat 2000 automatic processor. Exposure time was adjusted to 20–60 ms. Immobilization of the rats in prone position was achieved by placing them inside adjustable hand-made transparent plastic tubes, so that they could not move. Habituation to the recording chamber prior to commencement of the study did not significantly alter gastrointestinal motility (Cabezos et al., 2008). To further reduce stress, rats were released immediately after each shot (immobilization lasted for 1–2 min). X-rays were recorded at different times (immediately and 1, 2, 4, 6, and 8 h) after administration of the contrast medium. While taking the radiographs, the qualified investigator remained at least 2 m away from the X-ray source or behind a leaded wall, where radioactivity while shooting was not different from environmental readings. A trained investigator blind to the drug administered performed the analysis of the radiographs. Alterations in gut motility were semiquantitatively determined from the images by assigning a compounded value to each region of the gastrointestinal tract considering the following parameters: percentage of the gastrointestinal region filled with contrast (0–4); intensity of contrast (0–4); homogeneity of contrast (0–2); and sharpness of the gastrointestinal region profile (0–2). Each of these parameters was scored and a sum (0–12 points) was made. X-rays for characterization of vincristine and AM251 effects were obtained at HGUGM. X-rays to study the effect of AM630 were taken at URJC. Results were comparable for controls (triple administration of vehicle) obtained at both institutions.

The X-ray images were also analyzed with the aid of an image analysis system (Image J 1.38 for Windows, National Institute of Health, USA, free software: http://rsb.info.nih.gov/ij/) and the alterations of stomach size and caecum were studied.

### Compounds and drugs

Barium sulfate (Barigraf® AD, Juste SAQF, Madrid, Spain) was suspended in tap water and continuously hand-stirred until administration.

Vincristine was purchased from Sigma-Aldrich (Spain, experiments performed at HGUGM) or from Abcam (UK, experiments performed at URJC) and dissolved in saline.

N-(piperidin-1-yl)-5-(4-iodophenyl)-1-(2,4-dichlorophenyl)-4-methyl-1H-pyrazole-3-carboxamide (AM251; Gatley et al., 1996) and 6-iodo-2-methyl-1-[2-(4-morpholinyl)ethyl]-1H-indol-3-yl](4-methoxyphenyl) methanone (AM630; Hosohata et al., 1997) were purchased from Ascent Scientific Ltd (North Somerset, BS24 9 ES, UK).

Cannabinoid antagonists were dissolved in Tocrisolve, a commercially available water soluble emulsion composed of a 1:4 ratio of soya oil/water that is emulsified with the block co-polymer Pluronic F68 (Tocris, Cookson, Bristol, UK; 30 μl in 0.5 ml of saline solution).

### Statistical analysis

Data are presented as the mean values ± SEM. Differences between groups were analyzed using Student's *t*-test or two-way ANOVA followed by *post-hoc* Bonferroni multiple comparison test, as appropriate. Values of *p* < 0.05 were regarded as being significantly different.

## Results

### Histopathological effects of vincristine on intestinal tissues

**Figure 1:**
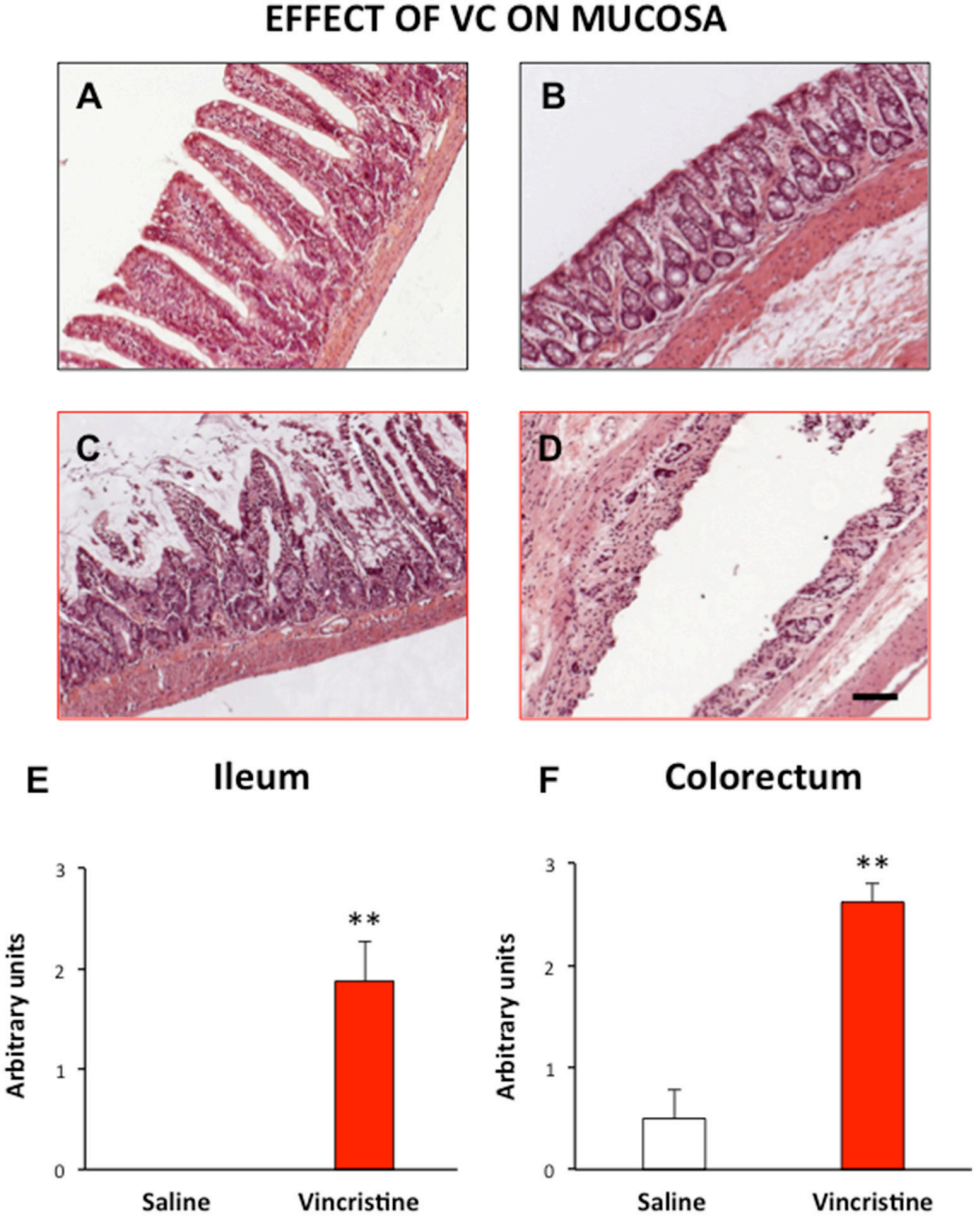
**Effect of vincristine treatment on the general structure of the rat intestine**. Rats were injected intraperitoneally with vincristine (0.5 mg/kg) or saline (1–2 ml/kg). Forty-eight hours after treatment, histological samples were embedded in paraffin, and stained with HE. Left row, small intestine: tissue samples from saline **(A)** and vincristine-treated animals **(C)** showing the damaged mucosa; quantitative analysis of the histological damage **(E)**. Right row, colorectum: tissue samples from saline **(B)** and vincristine-treated animals **(D)** showing the damaged mucosa; quantitative analysis of the histological damage **(F)**. Bars show mean values ± SEM for control (white) and vincristine-treated animals (red). Each group consisted of 4–8 rats. ^**^p < 0.01 vs. saline; (Student's *t*-test). Scale bar: 100 μm.

The histological pattern of the intestinal wall in HE stained sections is shown in Figure 1. A general and statistically significant damage was observed after vincristine administration. The epithelial layer was particularly affected, showing large areas with ulcers, and loss of normal architecture both in small (Figures 1A,B,E, *p* < 0.01) and large intestine (Figures 1C,D,F, *p* < 0.01). On the contrary, there were no differences regarding the extent of inflammatory nodules between vincristine-treated animals and controls.

### Effects of vincristine on gastrointestinal motor function

**Figure 2:**
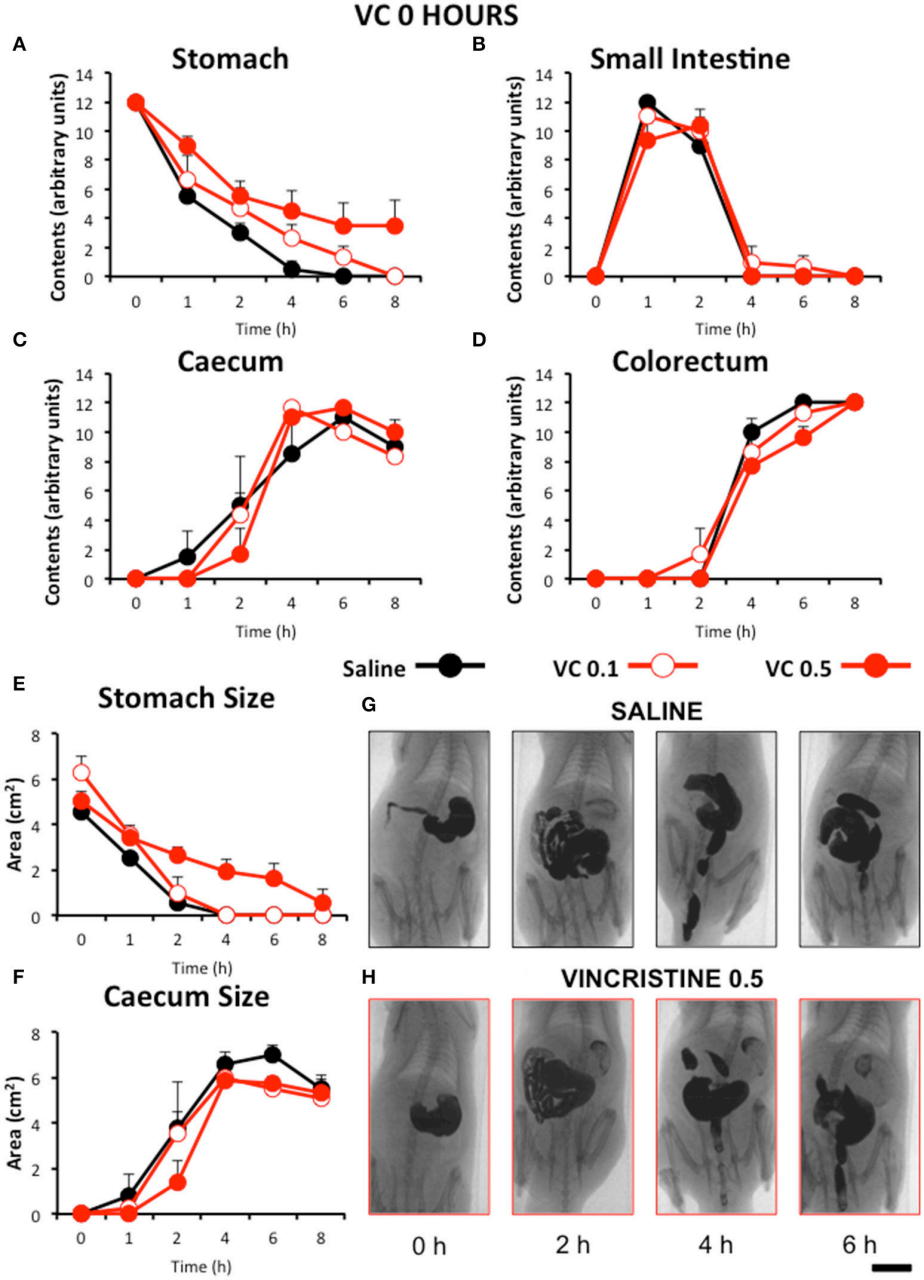
**Effect of vincristine immediately after administration on gastrointestinal motor function in the rat**. Gastrointestinal motor function was evaluated by radiological methods (see text) in: **(A)** stomach (gastric emptying); **(B)** small intestine; **(C)** caecum and **(D)** colorectum. Rats were injected intraperitoneally (i.p.) with: saline (1–2 ml/kg) or vincristine at 0.1 (VC 0.1) or 0.5 mg/kg (VC 0.5). Barium sulfate (2.5 ml, 2 g/ml) was intragastrically administered immediately after drug administration and X-rays were taken 0–8 h after. The size of stomach **(E)** and caecum **(F)** was determined with Image J. Data represent mean ± SEM (two-way ANOVA followed by *post-hoc* Bonferroni multiple comparison test). **(G,H)** Representative images of animals treated with saline and VC 0.5, taken at different times throughout the experiment. *n* = 8 each group. Scale bar: 23 mm.

**Figure 3:**
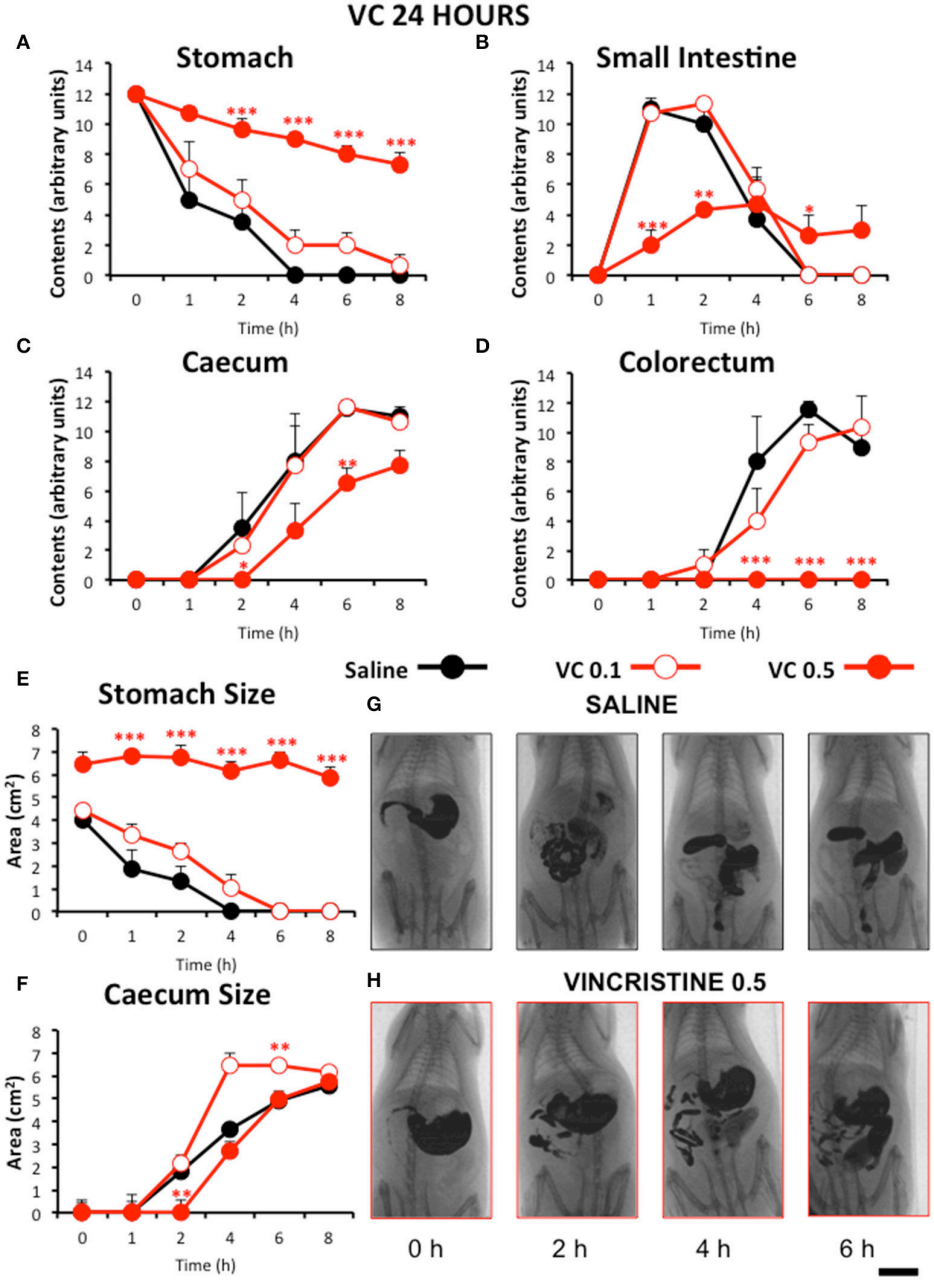
**Effect of vincristine 24 h after administration on gastrointestinal motor function in the rat**. Gastrointestinal motor function was evaluated by radiological methods (see text) in: **(A)** stomach (gastric emptying); **(B)** small intestine; **(C)** caecum and **(D)** colorectum. Rats were injected intraperitoneally (i.p.) with: saline (1–2 ml/kg) or vincristine at 0.1 (VC 0.1) or 0.5 mg/kg (VC 0.5). Barium sulfate (2.5 ml, 2 g/ml) was intragastrically administered 24 h after drug administration and X-rays were taken 0–8 h after. The size of stomach **(E)** and caecum **(F)** was determined with Image J. Data represent mean ± SEM. ^*^p < 0.05, ^**^p < 0.01, ^***^p < 0.001 vs. saline (two-way ANOVA followed by *post-hoc* Bonferroni multiple comparison test). **(G,H)** Representative images of animals treated with saline and VC 0.5, taken at different times throughout the experiment. *n* = 8 each group. Scale bar: 23 mm.

**Figure 4:**
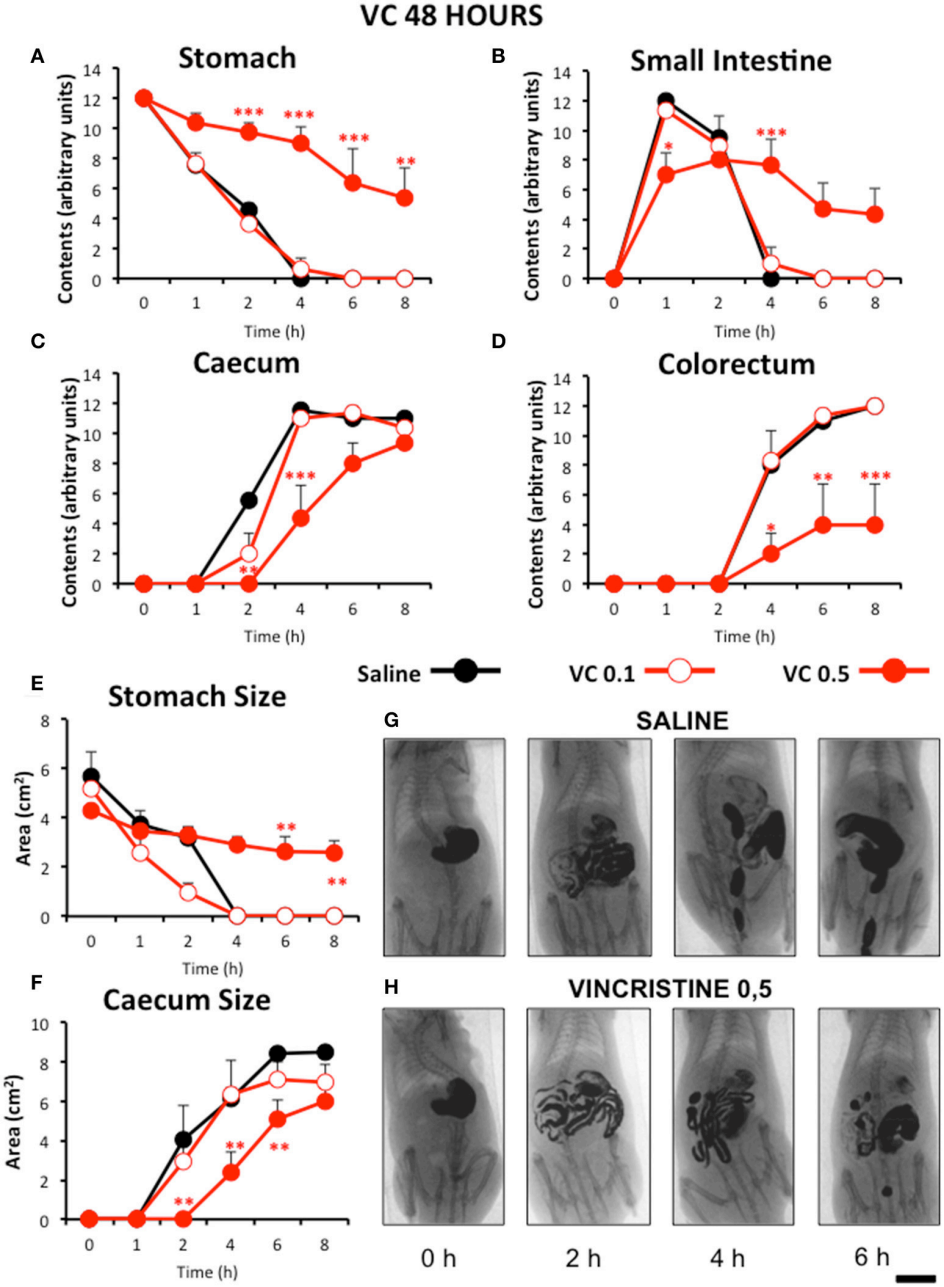
**Effect of vincristine 48 h after administration on gastrointestinal motor function in the rat**. Gastrointestinal motor function was evaluated by radiological methods (see text) in: **(A)** stomach (gastric emptying); **(B)** small intestine; **(C)** caecum and **(D)** colorectum. Rats were injected intraperitoneally (i.p.) with: saline (1–2 ml/kg) or vincristine at 0.1 (VC 0.1) or 0.5 mg/kg (VC 0.5). Barium sulfate (2.5 ml, 2 g/ml) was intragastrically administered 48 h after drug administration and X-rays were taken 0–8 h after. The size of stomach **(E)** and caecum **(F)** was determined with Image J. Data represent mean ± SEM. ^*^p < 0.05, ^**^p < 0.01, ^***^p < 0.001 *vs*. saline (two-way ANOVA followed by *post-hoc* Bonferroni multiple comparison test). **(G,H)** Representative images of animals treated with saline and VC 0.5, taken at different times throughout the experiment. *n* = 8 each group. Scale bar: 23 mm.

In control animals, when barium was given immediately after saline, gastric emptying was complete 4 h after barium. Barium content reached its maximum in the small intestine in just 1 h and it completely emptied into the caecum by 4 h. In most animals, barium started to stain the caecum and the colorectum 2 and 4 h after barium, respectively. Both organs filled progressively until the end of the study (Figure 2). When barium was given 24 (Figure 3) or 48 h (Figure 4) after saline, the motility curves were very similar to those obtained immediately after saline administration.

Compared with saline, acute administration of vincristine immediately before barium administration (0 h), did not induce any significant change on gastrointestinal motility, irrespective of the dose studied. Also, the quantitative analysis of the images did not show any significant change in the stomach or caecum size (Figure 2).

Remarkably, vincristine intensely and significantly reduced gastrointestinal motor function when this was radiographically evaluated 24 h after administration, but only at the dose of 0.5 mg/kg. In these animals, gastric emptying was progressive but at a much lower rate than in rats treated with saline or vincristine at 0.1 mg/kg (Figure 3A). Intestinal transit and filling of caecum were also delayed in vincristine-treated rats at the high dose (Figures 3B,C). Furthermore, at the end of the experiment (8 h), contrast had not reached the colorectal region in any of those animals (Figure 3D). These results were confirmed in the quantitative analysis of the images. In rats treated with vincristine at 0.5 mg/kg, the stomach size at the beginning of the experiment was increased compared to saline-treated animals and remained unchanged for the rest of the experiment (Figure 3E). In the caecum, vincristine had a dual effect; the lower dose of vincristine increased its size and the higher one reduced it (Figure 3F).

When serial X-rays were obtained 48 h after vincristine, gastrointestinal motor function was still decreased, although the effect was less pronounced than in the previous experiment. Gastric emptying and intestinal transit were reduced (Figures 4A,B), but gastric emptying started to recover 6 h after barium (54 h after vincristine), at least in some animals, and higher levels of barium contents were reached in the small intestine at all time-points. The effect on caecum was similar to the previous experiment, but that on the colorectum was less intense and fecal pellets were seen in some animals (Figures 4C,D). These results were confirmed in the quantitative analysis. Thus, 0 h after contrast (48 h after vincristine), the stomach size was again comparable to that in the saline group, but not much further change was apparent in this region (Figure 4E). Vincristine reduced the size of the caecum at the highest dose used (Figure 4F).

### Effect of the cannabinoid antagonists on ileus induced by vincristine

**Figure 5:**
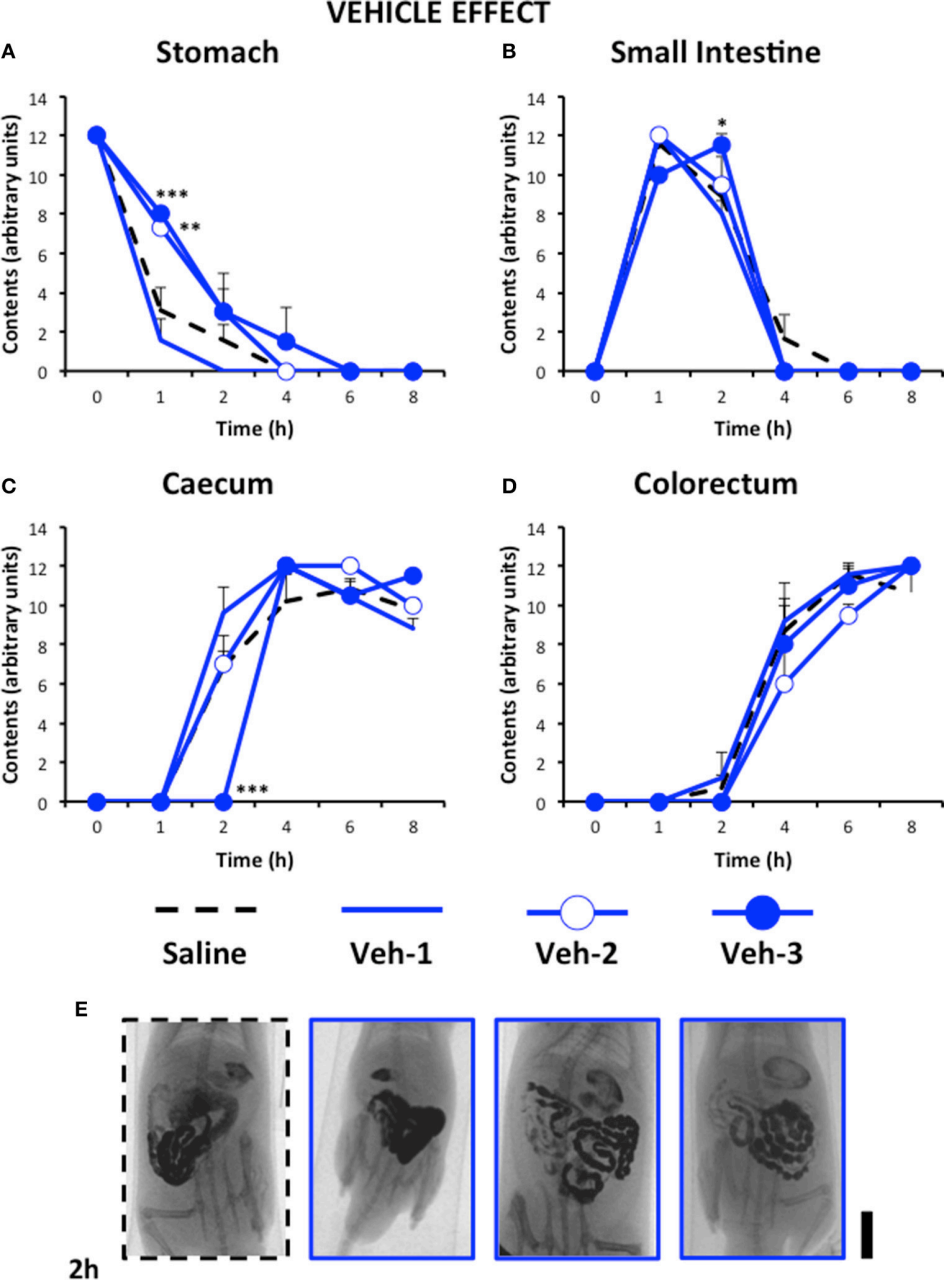
**Effect of saline or the cannabinoid vehicle on gastrointestinal motor function in the rat**. Gastrointestinal motor function was evaluated by radiological methods (see text) in: **(A)** stomach (gastric emptying); **(B)** small intestine; **(C)** caecum and **(D)** colorectum. Rats were injected intraperitoneally (i.p.) with: saline (1–2 ml/kg) or the cannabinoid vehicle once (20 min before saline, Veh-1), twice (before and 24 h after saline, Veh-2), and thrice (before, 12 and 24 h after saline, Veh-3). Barium sulfate (2.5 ml, 2 g/ml) was intragastrically administered immediately or 24 h after drug administration and X-rays were taken 0–8 h after. Data represent mean ± SEM. ^*^p < 0.05, ^**^p < 0.01, ^***^p < 0.001 vs. saline (two-way ANOVA followed by *post-hoc* Bonferroni multiple comparison test). *n* = 4–8 animals per group. **(E)** Representative images of saline- and Veh-treated animals 2 h after contrast. Scale bar: 23 mm.

Figure 5 shows the motility curves for controls used in this experiment. In addition to the effect of saline, the effect of injecting the cannabinoid vehicle once (20 min before saline), twice (before and 24 h after saline) and thrice (before, 12 and 24 h after saline) is shown. As can be seen, injecting the vehicle only once did not produce any effect compared to saline-treated animals. However, when it was injected twice or thrice, significant delays in gastric emptying and in filling of small intestine and caecum were seen. For the following experiments, the effects of the different drugs are compared to those of the vehicle given thrice, since it was the pattern which induced more changes compared to saline (although still very different to that found in vincristine-treated animals, see below).

**Figure 6:**
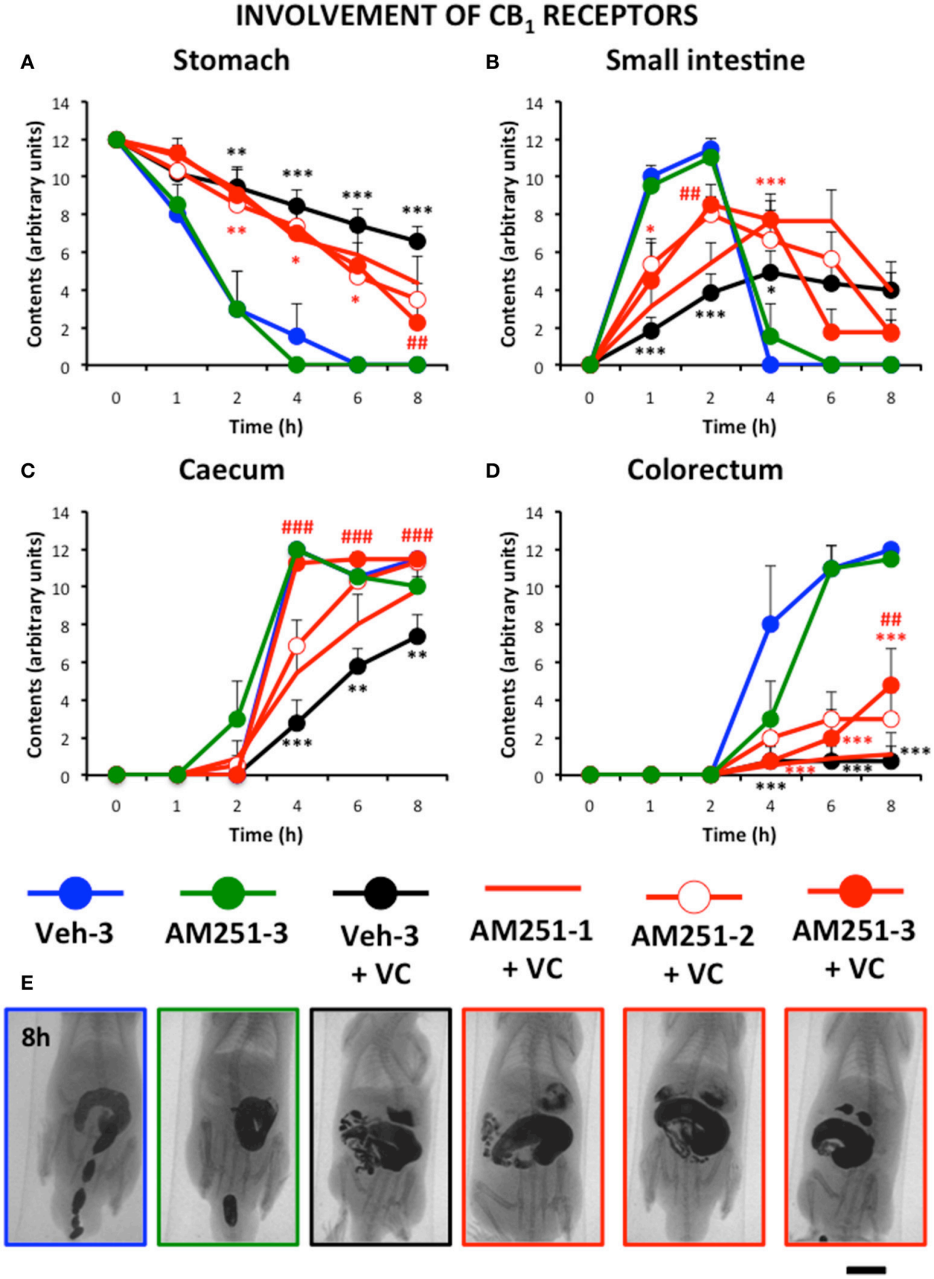
**Effect of the CB_1_ antagonist AM251 on the alterations induced by vincristine on gastrointestinal motor function in the rat**. Gastrointestinal motor function was evaluated by radiological methods (see text) in **(A)** stomach (gastric emptying); **(B)** small intestine; **(C)** caecum and **(D**) colorectum. Rats received two intraperitoneal injections (i.p.). One was saline (1–2 ml/kg) or vincristine at 0.5 mg/kg (VC). The other one was the cannabinoid vehicle given three times (Veh-3) or AM251 given once (20 min before saline or vincristine: AM251-1), twice (before and 24 h after saline or vincristine: AM251-2), or thrice (before, 12 and 24 h after saline or vincristine: AM251-3). Barium sulfate (2.5 ml, 2 g/ml) was intragastrically administered 24 h after saline or vincristine administration and X-rays were taken 0–8 h after. Data represent mean ± SEM. ^*^p < 0.05, ^**^ *p* < 0.01, ^***^p < 0.001 vs. Veh-3; ##*p* < 0.01; ###*p* < 0.001 vs. Veh 3 + VC (two-way ANOVA followed by *post-hoc* Bonferroni multiple comparison test). *n* = 4–8 animals each group. **(E)** Representative images of the different treatments 8 h after contrast. Scale bar: 23 mm.

Figure 6 shows that the CB_1_ antagonist AM251 (1 mg/kg) improved gastrointestinal motor function compared to vincristine-treated rats and that this effect increased with the number of times it was injected (3 > 2 > 1). However, the normalizing effect of AM251 was different in each gastrointestinal region. Thus, in the stomach and colorectum, AM251 given thrice exerted a significant but relatively small effect. In contrast, it almost normalized the motility curve in the small intestine, and completely normalized the curve in the caecum. Efficacy of AM251 was lower in animals treated with the compound only twice and even lower when it was administered only once. AM251 given thrice did not exert any significant effect compared to its vehicle given also thrice.

**Figure 7:**
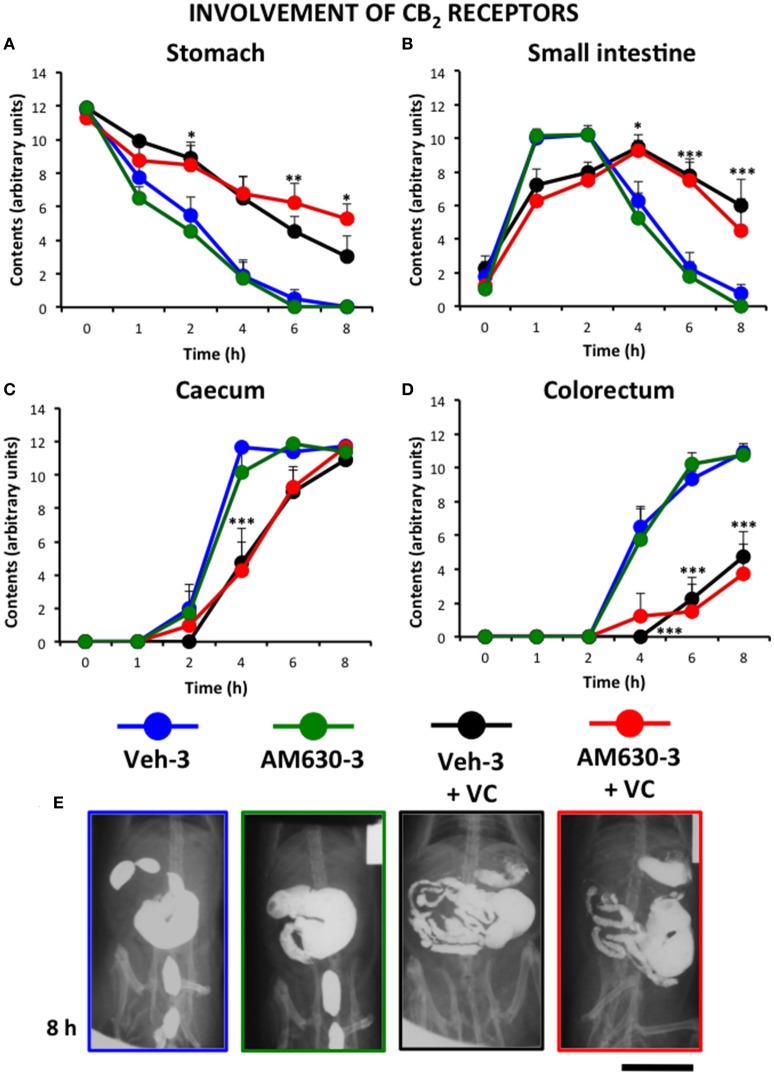
**Effect of the CB_2_ antagonist AM630 on the alterations induced by vincristine on gastrointestinal motor function in the rat**. Gastrointestinal motor function was evaluated by radiological methods (see text) in: **(A)** stomach (gastric emptying); **(B)** small intestine; **(C)** caecum and **(D)** colorectum. Rats received saline (1–2 ml/kg) or vincristine at 0.5 mg/kg (VC). The cannabinoid vehicle (Veh-3) or AM630 (AM630-3) were administered thrice (before, 12 and 24 h after saline or vincristine administration). Barium sulfate (2.5 ml, 2 g/ml) was intragastrically administered 24 h after saline or vincristine administration and X-rays were taken 0–8 h after. Data represent mean ± SEM. ^*^p < 0.05, ^**^p < 0.01, ^***^p < 0.001 vs. Veh-3 (two-way ANOVA followed by *post-hoc* Bonferroni multiple comparison test). *n* = 8 each group. **(E)** Representative images of the different treatments 8 h after contrast. Scale bar: 30 mm.

Finally, the effect of the selective CB_2_ antagonist AM630 was tested. For ethical reasons, and due to the fact that the CB_1_ antagonist showed the best results after its triple administration, we only used this pattern of administration to test the effect of AM630. In high contrast with the effect of AM251, the triple administration of AM630 did not significantly modify the effect of vincristine (Figure 7).

## Discussion

This is the first work in which vincristine-induced gastrointestinal dysmotility has been characterized using radiographic methods in experimental animals. In addition, we have demonstrated that the selective CB_1_ cannabinoid antagonist AM251 (but not AM630, a CB_2_ selective antagonist), is capable of reducing the effect of vincristine, suggesting that an increased cannabinoid tone is, atleast partially, responsible for the alterations induced by this antitumoral drug on gastrointestinal motor function.

### Vincristine effect on gastrointestinal motor function in the rat

Gastrointestinal dysmotility associated to vinca alkaloids, including vincristine, is a known cause of drug-induced gastrointestinal toxicity (Bradley, 1968). Here, vincristine did not significantly alter gastrointestinal motility at a low (0.1 mg/kg) or high dose (0.5 mg/kg) when the X-ray study was performed immediately after its administration. However, when the radiographic study was carried out 24 or 48 h after the antitumoral drug at 0.5 mg/kg, an intense and significant decrease in gastric emptying and intestinal transit was observed. These results indicate that vincristine-induced dysmotility may need a relatively long time to occur or that higher doses might be needed to see early effects of this drug on gastrointestinal motor function. We did not increase the dose or the observation time because the higher dose used here was similar to the LD_50_ in rats and mortality associated to this dose may occur 4–6 days after its administration (Uy et al., 1967).

Several previous investigations have reported that vincristine-induced gastric hypomotility is not an early event (Kaneko et al., 2001; Tsukamoto et al., 2011). The delayed effect of vincristine contrasts with that of other antineoplastic drugs, like cisplatin, which provokes gastric dysmotility within a much shorter time in rats (Cabezos et al., 2008, 2010; Vera et al., 2014). Cisplatin immediate (acute) effect on gastric motor function is due to serotonin release and vagal activation, through the stimulation of 5-HT_3_ receptors (Vera et al., 2014), and underlies its intense emetogenic effect in experimental animals (Holmes et al., 2009; du Sert et al., 2011; Horn, 2014) and humans (Navari, 2013). Cisplatin may also produce “delayed” emesis in humans, and we observed “delayed” gastric dysmotility and pica, a surrogate marker of nausea in rodents (Takeda et al., 1993), after cisplatin administration in the rat (Cabezos et al., 2008). Cisplatin-induced delayed emesis seems to be more dependent upon other mechanisms, including the activation of NK_1_ receptors through the release of substance P (Navari, 2013; Rudd et al., 2016). These mechanisms justify the usefulness of 5-HT_3_ and NK_1_ antagonists for prevention of emesis associated to highly emetogenic chemotherapy in cancer patients (Navari, 2013). Vincristine may induce nausea and emesis in dogs and humans, but the incidence and intensity of these effects are much lower than with cisplatin (Navari, 2013; Mason et al., 2014) and the mechanisms might be different (see below).

The altered motility curve observed here for the small intestine 24 h after vincristine may be due, at least partly, to the delayed gastric emptying (barium reached the small intestine much later in vincristine- than in saline-treated animals). However, this would probably have produced a motility curve for the small intestine very similar in shape to that in control animals, but displaced to the right (this occurred after acute cisplatin, which only alters gastric motility; Cabezos et al., 2008). In the present study, the curve was completely distorted, suggesting that vincristine produced direct effects in this region. Direct effects of vincristine in the small intestine might include altered myoelectric activity, increased tone and spasmogenic actions, as previously suggested (Sharma, 1979, 1988; Sninsky, 1987). Small intestinal transit was accelerated in rats in the first few hours after vinblastine (Sharma, 1979), but we did not detect such an effect of vincristine in our non-invasive study. In fact, the motility curve of caecum looked very similar (parallel) to that in control animals both 24 and 48 h after vincristine (0.5 mg/kg), but displaced to the right, further suggesting that small intestinal transit was delayed. Interestingly, in spite of the fact that caecum filled adequately, there was a complete absence of stained fecal pellets in vincristine-treated rats for the whole duration of the radiologic study when it was performed 24 h after the antitumoral drug, suggesting that vincristine directly suppressed motility in colorectum, which is in accordance with the reports of constipation associated to treatment with vinca alkaloids, in both animals and humans (Harris and Jackson, 1977; Garewal and Dalton, 1985; Ikehara, 1992; Leker et al., 1997; Chae et al., 1998; Wang et al., 2000; Essa et al., 2014; Yasu et al., 2016).

Several factors may contribute to the effects found in the stomach, small intestine, caecum and colorectum 24–48 h after vincristine. Chemotherapy-induced gastrointestinal toxicity can be caused by direct damage to mucosal epithelial cells or by stimulation of the vomiting center or chemoreceptor trigger zone (Kaneko et al., 2001). Vincristine is known to induce metaphase arrest, severe villous atrophy and mucosal erosions (Beró and Jávor, 1985), which we found in our histological study. This effect would disrupt the intestinal barrier function and could contribute to dysmotility. In contrast, we did not observe evident changes in the presence of inflammatory cells within the gut wall, suggesting that these might not be determinant to acute vincristine-induced dysmotility, although this must be systematically studied. According to previous reports, direct effects on the smooth muscle layers (Kaneko et al., 2001) or the possible influence of enhanced adrenergic activity due to neuropathic pain (Peixoto Júnior et al., 2009) seem unlikely. Interestingly, due to the known neurotoxicity of the compound (whose direct effect on the vomiting center to induce gastric dysmotility and emesis cannot be discarded) and to several functional and histological evidences, the development of an autonomic neuropathy has been suggested to contribute to vincristine-induced gastrointestinal hypomotility, particularly after high doses or chronic treatments (Smith, 1967; Peixoto Júnior et al., 2009). However, a systematic analysis of the possible changes in structure and in marker expression in the enteric nervous system after vincristine treatment, as those performed with other antineoplastic drugs (Vera et al., 2011; Wafai et al., 2013; McQuade et al., 2016), is still required to define the precise role of neuropathy affecting the enteric nervous system, particularly the myenteric plexus, on gastrointestinal motor disturbances induced by vincristine. This might be particularly evident in chronic treatments.

Importantly, gastrointestinal ileus induced by vincristine, in contrast to sensory neuropathy, seems to be transient and is reverted soon after treatment discontinuation (Sharma, 1988; Chae et al., 1998; Peixoto Júnior et al., 2009). However, in addition to reducing quality of life, vincristine-induced gastrointestinal dysmotility may be problematic and even fatal, particularly under certain circumstances (liver failure, concomitant condition predisposing to constipation, drug interactions, or even accidental overdose: (Toghill and Burke, 1970; Leker et al., 1997; Bermúdez et al., 2005; Uner et al., 2005; Levêque et al., 2009; Diezi et al., 2010; Le Guellec et al., 2012; Essa et al., 2014; Yasu et al., 2016). This justifies the search for anti-ileus treatments.

Very few agents have been tested in vincristine-induced gastrointestinal ileus and most references are case reports with low numbers of subjects (Harris and Jackson, 1977; Jackson et al., 1982; Garewal and Dalton, 1985; Ikehara, 1992; Tsukamoto et al., 2011; Essa et al., 2014; Mason et al., 2014). To the best of our knowledge, the possible role of cannabinoids has never before been tested either in experimental animals or in the clinic.

### Role of cannabinoids on gastrointestinal ileus induced by vincristine

Cannabinoids have been used empirically and traditionally to treat different disorders including those of the gut, ranging from enteric infections and inflammatory conditions to motility alterations, emesis, and abdominal pain (Izzo and Camilleri, 2009; Izzo and Sharkey, 2010; Abalo et al., 2012; Abalo and Martín-Fontelles, 2017; Salaga et al., 2017; Vera et al., 2017). Central and peripheral cannabinoid receptors seem to be involved in the regulation of gastrointestinal motility. Cannabinoid CB_1_ receptors are mainly found in nervous cells, including those of the myenteric plexus (Abalo et al., 2012; Vera et al., 2017), principal responsible for intestinal motility. Interestingly, these gastrointestinal CB_1_ receptors appear to exert a tonic control over the enteric nervous system, and operate as a “brake” for neural over-reactivity (Schicho and Storr, 2011; Abalo et al., 2012). In fact, agonists acting at CB_1_ receptors may potently depress gastrointestinal motor function even in the absence of significant central effects (Abalo et al., 2015). In contrast, CB_2_ receptors are mainly found in immune cells, and have anti-inflammatory effects (Turcotte et al., 2016). Normally, CB_2_ agents do not alter gastrointestinal motor function (Abalo et al., 2009, 2010, 2011, 2015). However, it has been shown that CB_2_ receptors are overexpressed in the myenteric neurons under inflammatory conditions, and in such cases, they may also reduce transit and normalize intestinal motor function (Mathison et al., 2004; Duncan et al., 2008; Wright et al., 2008). Thus, selective CB_1_ and CB_2_ cannabinoid receptor antagonists may be useful in situations in which gastrointestinal motor function is reduced.

The effect of CB_1_ antagonists on motility in control animals is to some extent controversial, with some reports showing increased transit (Mathison et al., 2004) and others showing no effects (Landi et al., 2002), suggesting that the gastrointestinal cannabinoid tone may be sensitive to slight differences in experimental conditions (Abalo et al., 2009, 2010, 2011, 2015). In humans, diarrhea was present in some obese patients treated with rimonabant and other cannabinoid antagonists (Waterlow and Chrisp, 2008). In animal models of paralytic ileus, CB_1_ receptor was overexpressed and anandamide levels were increased (Mascolo et al., 2002; de Filippis et al., 2008). Thus, an increased cannabinoid tone, due to released endocannabinoids and/or CB_1_ overexpression, seem to be involved in the development of paralytic ileus and strategies aimed at normalizing endocannabinoid levels/tone could be therapeutically useful in these conditions. Consequently, the CB_1_ selective antagonist AM251 (with *IC*_50_ = 8 nM, *K*_i_ = 7.49 nM, and 306-fold selectivity over CB_2_ receptors, Lan et al., 1999) was used here to see if vincristine effects are mediated by a similar mechanism.

In our study, AM251, at a dose that lacked any significant effect on GI motility in control animals (1 m/kg), reduced the effect of vincristine on gastric emptying and intestinal transit. This was achieved when the antagonist was administered twice (once every 24 h) or thrice (once every 12 h). The gastrointestinal region most sensitive to the effect of the CB_1_ antagonist was the small intestine, and transit was close to normal after its triple administration, as suggested by the motility curves for the small intestine and, even more, for the caecum, which showed normal filling. In contrast, altered gastric emptying and colorectal motility after AM251 triple administration only partially improved at the end of the study (8 h after contrast, 32 h after vincristine). Thus, an increase in cannabinoid tone affecting CB_1_ receptors might underlie some of the effects of vincristine in the stomach and colorectum, but other factors may be more influential in these regions, whereas vincristine-induced small intestinal ileus seems to depend mostly, if not completely, on increased CB_1_ receptor activity. Accordingly, in LPS-induced septic models of ileus, AM251 increased myoelectric activity of rat jejunum *in vitro* and upper gastrointestinal transit in mice (measured with the charcoal method; Li et al., 2010). Rimonabant (another CB_1_ antagonist receptor) alleviated gastrointestinal symptoms in a murine model of paralytic ileus induced by intraperitoneal injection of acetic acid, modeling peritonitis, and an anandamide uptake inhibitor worsened motility even further (Mascolo et al., 2002). On the other hand, in a model of postoperative ileus, upper gastrointestinal transit was similarly reduced in wild type and knock-out mice for CB_1_ receptors (although the inflammatory response was more intense in the latter), suggesting that altered motility in this model might not be necessarily or only due to increased CB_1_ receptor activation (Li et al., 2013a).

Since AM251 (and rimonabant) is considered both an antagonist and an inverse agonist, at this stage it is not clear if our results are due to an increased basal activity of CB_1_ receptors after vincristine, linked to overexpression, and/or to the release of endocannabinoids. As mentioned above, increased anandamide levels were found to occur in models of paralytic ileus (Mascolo et al., 2002), and expression of CB_1_ receptors was increased in different models of ileus (Mascolo et al., 2002; de Filippis et al., 2008). Interestingly, the motility curves obtained from vincristine-treated animals here were very similar to those previously obtained from control animals treated with cannabinoids, whose effects were dependent upon CB_1_ activation and much more potent on intestinal regions (particularly the small intestine) than on the stomach (Abalo et al., 2009, 2010, 2015), suggesting that the release of endogenous cannabinoids might be involved in vincristine effects.

AM630 did not significantly modify the effect of vincristine on gut motility. The involvement of CB_2_ receptors in experimental models of ileus is less clear than that of CB_1_ receptors. Thus, in models of septic ileus, some researchers described that inactivation of either CB_1_ or CB_2_ receptors normalized jejunal myoelectric activity and upper gastrointestinal transit (Li et al., 2010). In contrast, inactivation of CB_2_ receptors did not normalize reduced motility associated to intraperitoneal acetic acid administration (Mascolo et al., 2002).

Finally, it cannot be discarded that AM251 exerted its effects through another mechanism. Interestingly, it has been described as a GPR55 agonist (*EC*_50_ = 39 nM; Henstridge et al., 2010). As mentioned above, when used alone in control animals, gastrointestinal motility was not significantly altered, suggesting that GPR55 receptors were not activated in these animals. O-1602, another agonist of GPR55 receptors (but 3-fold more potent than AM251 upon them: *EC*_50_ = 13 nM), did not alter upper gastrointestinal transit when used at 10 mg/kg in control or LPS-treated mice (which showed reduced transit), whereas cannabidiol, which is considered a GPR55 antagonist, counteracted O-1602, and LPS-effect (Lin et al., 2011; Li et al., 2013b). Thus, if GPR55 was overexpressed by vincristine treatment, as by LPS, it is more likely that a GPR55 antagonist was more useful to counteract GPR55 overactivation than a GPR55 agonist like AM251. The involvement of GPR55 receptors in vincristine-induced dysmotility will be specifically investigated in future work.

In conclusion, the fact that AM251 (but not AM630) is capable of reducing the effect of vincristine suggests that, like in other experimental models of paralytic ileus, an increased cannabinoid tone acting through CB_1_ receptors is, at least partially, responsible for the alterations induced by vincristine on gastrointestinal motor function. The combination of different technniques, including immunohistochemistry (to locate the cells expressing the receptors) and molecular biology (to determine the levels of receptors and ligands, if appropriate), will help determine the precise mechanism of action involved in AM251 effect. Whatever this may be, ours is a clinically relevant finding and encourages the exploration of strategies aimed at reducing CB_1_ receptor activity to prevent or palliate vincristine-induced ileus in the clinic.

## Author contributions

RA designed the study. GV, AL, RG, and JU performed the experiments and analyzed the data. RA and GV wrote the manuscript. MM contributed financial support and essential intellectual input. All authors reviewed and approved the final version of the manuscript.

## Funding

This work was supported by Ministerio de Ciencia e Innovación (SAF2009-12422-C02-01, SAF2012-40075-C02-01), Universidad Rey Juan Carlos—Comunidad de Madrid (URJC-CM-2006-BIO-0604) and Comunidad de Madrid (S-SAL/0261/2006; S2010/BMD-2308).

### Conflict of interest statement

The authors declare that the research was conducted in the absence of any commercial or financial relationships that could be construed as a potential conflict of interest.
